# Laser Ultrasound Inspection Based on Wavelet Transform and Data Clustering for Defect Estimation in Metallic Samples

**DOI:** 10.3390/s19030573

**Published:** 2019-01-30

**Authors:** Hossam Selim, Miguel Delgado Prieto, José Trull, Luis Romeral, Crina Cojocaru

**Affiliations:** 1 Physics Department, Universitat Politècnica de Catalunya, Rambla Sant Nebridi 22, 08222 Terrassa, Barcelona, Spain; hossam.eldin.mohamed.selim@upc.edu (H.S.); jose.francisco.trull@upc.edu (J.T.); crina.maria.cojocaru@upc.edu (C.C.); 2 Electronic Engineering Department, Universitat Politècnica de Catalunya, Rambla Sant Nebridi 22, 08222 Terrassa, Barcelona, Spain; luis.romeral@upc.edu

**Keywords:** laser ultrasound, damage visualization, spectral signal processing, ultrasound propagation, time of flight, wavelet transform, non-destructive testing

## Abstract

Laser-generated ultrasound is a modern non-destructive testing technique. It has been investigated over recent years as an alternative to classical ultrasonic methods, mainly in industrial maintenance and quality control procedures. In this study, the detection and reconstruction of internal defects in a metallic sample is performed by means of a time-frequency analysis of ultrasonic waves generated by a laser-induced thermal mechanism. In the proposed methodology, we used wavelet transform due to its multi-resolution time frequency characteristics. In order to isolate and estimate the corresponding time of flight of eventual ultrasonic echoes related to internal defects, a density-based spatial clustering was applied to the resulting time frequency maps. Using the laser scan beam’s position, the ultrasonic transducer’s location and the echoes’ arrival times were determined, the estimation of the defect’s position was carried out afterwards. Finally, clustering algorithms were applied to the resulting geometric solutions from the set of the laser scan points which was proposed to obtain a two-dimensional projection of the defect outline over the scan plane. The study demonstrates that the proposed method of wavelet transform ultrasonic imaging can be effectively applied to detect and size internal defects without any reference information, which represents a valuable outcome for various applications in the industry.

## 1. Introduction

Embedded structural damage is a typical defect in metallic parts and structures exposed to a defective manufacturing procedure or subjected to a complex and cyclic loading during their service. Among various non-destructive evaluation (NDE) and structural health monitoring (SHM) techniques, ultrasound-based approaches have been widely applied in the last decade for metallic materials inspection [1,2]. In fact, acquiring inspection data provides information for quality control, condition-based maintenance, and preventive measures that are all related to industrial safety, reliability, and quality features [3,4]. 

The emitter-receiver ultrasonic inspection scheme, based on ultrasonic transducers, has been classically adopted for damage detection and failure localization as a cost-effective NDE strategy [5,6]. However, the low output excitation power of the ultrasonic transducers prevents such approaches from being used remotely. In this regard, non-contact variants of the ultrasonic excitation source have been investigated in recent years [7]. However, the acoustic coupling impedances limit their applicability to use in air at an airgap of few millimeters or to use in water following water immersion procedures [8,9].

In recent years, as an alternative to exclusive ultrasonic approaches, photonic strategies, based on laser-induced ultrasound, have been revealed as a powerful remote NDE technique. These approaches offer the possibility of inspection at higher resolution ratios, even when the power density of the pulsed laser is lower than the ablation threshold of the material under test, i.e. working on the thermo-elastic regime [10]. Hence, the electromagnetic radiation carried by the laser excitation pulse is rapidly absorbed into a shallow volume of the metallic material, and creates a localized heating effect resulting in a thermo-elastic expansion, finally inducing a stress pulse that generates a multi-component ultrasound wave [11,12,13,14,15,16,17]. Although complete laser-based technologies, such as optical interference or holographic interferometry, can be used as well for crack detection of materials, the related inspection setup is highly sensitive to stability and environmental conditions, which mostly limits this strategy to laboratory procedures [18,19]. In this regard, the hybridization of the laser-ultrasonic technique, taking advantage of the optical system for ultrasonic wave generation, and conventional ultrasonic transducers for detection, is currently being investigated to overcome the drawbacks of the aforementioned approaches.

Indeed, this approach allows ultrasonic wave generation at a far distance from the object, enabling remote testing without the need for a direct contact with the excitation source. The resulting broad excitation frequency bandwidth covers the majority of the ultrasonic bandwidths of interest for available applications involving material characterization. Moreover, the ultrasound transducers for reception, already integrated in the structure following common SHM approaches or strategically positioned in the component under inspection, may cover the measuring of resulting responses [20]. Research on such a laser-ultrasonic inspection scheme has been done by different authors. For example, W. Zeng et al. showed that the presence of damage in the component under inspection can cause a significant increase of amplitude and bandwidth of reflected wave signals. In this regard, the Wigner-Ville transform was proposed for quantization [21]. However, the procedure requires the selection of a specific frequency component to track the propagation of the ultrasonic wave and the damage effects. Also, J.-R. Lee et al. presented the study of a laser ultrasonic system designed for the damage visualization of a target structure located at a large distance [22]. Although the work showed excellent application feasibility, the study is focused on surface defects. Another significant work is the one proposed by B. Park et al., where the resulting ultrasonic waves in the presence of damage, were studied and a correlation strategy among multiple measurements was proposed to enhance characteristic damage effects in ultrasonic images [23]. C. Pei et al., studied the interaction process of the laser generated ultrasound with internal defects. He developed a method to characterize defect size based on time of flight (TOF) analysis of the scattered waves [24].

Although these procedures show good qualitative results over rotating surfaces, the damage quantization is limited. The detection and quantization of embedded damages in metallic components is still a challenging field of research in terms of applying suitable signal processing techniques and defect reconstruction methodologies to extract characteristic ultrasonic wave contents and generate reliable defect estimations. In this regard, time-domain analysis represents a set of powerful tools which are applied often for damage detection purposes [5]. If the amplitude of the ultrasonic signal events exceeds a certain threshold, they are recorded and characterized. Although these methods are used even for damage localization, their intrinsic limitations restrict them to certain applications such as fault detection, since the coexistence of multiple frequency-modes may mask characteristics patterns for damage reconstruction [25]. Thus, many studies fall into the frequency-domain analysis of ultrasonic waves [26]. However, the Fourier transform does not provide information about the evolution over time of each component and its instantaneous strength, which is critical in dealing with ultrasonic propagation modes and damage effect characterization. The Fourier transform may even mask the components that appear in a given time instant, but they are of very short duration [27]. In consequence, different time-frequency domain transforms are the most promising techniques to apply for ultrasonic signals. Some authors, such as Y. Zhang et al. [28], studied Empirical Mode Decomposition (EMD) to analyze the ultrasonic signals captured from an object that suffers from a certain defect. Afterwards, the Fourier transform was applied over the selected Intrinsic Mode Functions (IMFs). The study exhibits good characterization capabilities, but the number of the resulting IMFs cannot be controlled a priori. Other authors focused their efforts on comparing and clarifying the capabilities of different Cohen’s class-based time-frequency distributions [29]. The main drawbacks of these methods are related to time and frequency resolutions and undesirable components called cross-terms, which appear in the time-frequency representation and make interpretation difficult. In this regard, the Wavelet Transform (WT) is considered a suitable time-frequency technique dealing with ultrasonic signal analysis since it is based on the correlation between the target signal and a shifted set of dilated and compressed versions of a specific wavelet [30]. The flexibility of choosing the proper mother wavelet is one of the strongest advantages of using WT, since the choice of the mother wavelet for a particular problem instead of classical sinus based, may improve characteristic pattern identification. This fact allows that, using the proper mother wavelet, dramatic signal-to-noise ratios can be obtained, which represents a huge potential for ultrasonic signal analysis and defect pattern identification [31,32]. Although some studies have exhibited the potential of the WT to analyze ultrasonic signals, the analysis and interpretation of the resulting time-frequency maps are still a challenge in the field. In fact, the energy values extracted after the time-frequency calculation are commonly used for a qualitative interpretation (i.e., ultrasonic wave arrival time), but they are not further analyzed in order to extract a damage indicator containing the diagnosis information itself. What is more challenging is a quantitative damage visualization methodology, as proposed in this study.

In this paper, a defective metallic component for damage detection and visualization through a laser-ultrasonic approach is studied. For this purpose, the wavelet transform is proposed as a time-frequency processing tool. The characteristic damage frequency patterns are studied, identified, and tracked back to the boundaries of the source, i.e., the damage spatial location in the component under inspection. Then, a damage reconstruction procedure is proposed based on the estimated time of flights of the ultrasonic damage propagation patterns. The contribution of this study lies in providing a new approach of damage detection, localization, and shape reconstruction based on detection and processing of the damage ultrasonic echo characteristics. An adaptive resolution method using wavelet transform was chosen in order to check the proposed methodology under a laser-ultrasonic scheme.

Novelties of this work include a new damage pattern tracking method over the WT-based time-frequency maps, and a time of flight-based damage localization and visualization procedure; both are supported by the application of a density-based spatial clustering algorithm in order to identify the highest probability regions of damage location. It must be noticed that for the first time, to the authors’ knowledge, this processing scheme and this damage visualization procedure have been used in the laser-ultrasonic inspection of metallic specimens. According to the obtained results, the proposed methodology is reliable and feasible for defect detection and quantification of metallic damages in industrial applications since the proposed method does not require any reference information.

This paper is organized as follows: the theoretical basis and its suitability for ultrasound processing of the wavelet transform are presented in Section 2. The materials and method, including the experimental setup, are explained in Section 3. The competency of the method and the experimental results are presented and discussed in Section 4. Finally, the paper shows the conclusion dissemination in Section 5.

## 2. Wavelet Transform

Spectral analysis of A-scan signals is very important to get information about both time and frequency responses of the signal in the same diagram. Many time-frequency transforms are available for this purpose. One of the most famous related algorithms is the Short-Time Fourier Transform (STFT). It simply applies the Fourier Transform approach to the measured signal multiplied by a non-zero function for a short-time window.

By shifting these time windows across the whole temporal axis of the measured signal, we obtain the spectrogram of the signal with spectral data at each time. One major disadvantage of this technique is the fact that the time window is fixed during the whole analysis. This fixed window results in a high resolution only in either the frequency or the temporal spectrum, i.e., a wider window results in a better frequency resolution with a poor time resolution and vice versa for a shorter window. Other techniques that use non-parametric and bilinear transforms are also good examples of these spectral analysis topologies that overcome the disadvantages of STFT. They can be expressed generally as Cohen’s class distribution functions that generate a function representing intensity per time and per frequency [29]. The output of such time-frequency distribution is a 2D time-frequency diagram that provides important information for time and frequency components called auto-terms.

There are other irrelative and undesirable components defined as cross-terms that are oscillating components found between the auto-terms. Cross-terms hide some information of the main signal and reduce the resolution, making it difficult to interpret the resulting distribution [29,33]. An alternative approach to the STFT, to overcome the constant resolution problem, and for the Cohen’s class distributions to overcome cross-terms overlapping, is the wavelet transform. The WT uses small and finite oscillating signals called wavelets as a basic function for representing a signal. The wavelet-based transform has the advantage of adapting time and frequency resolutions to the signal contents, which is known as multi-resolution analysis. This results in a good time-resolution and a limited frequency-resolution at high frequencies, and a good frequency-resolution and a limited time-resolution at low frequencies, which is highly suitable for characterizing short-duration events of high frequencies and long-duration events of low frequency components [34].

The main purpose of the initially selected mother wavelet is to provide a source function to generate the daughter wavelets which are simply the translated and scaled versions of the mother wavelet [35]. The WT employs a sliding window function that is used to decompose the target signal into a sum of wavelets added together. Each wavelet has finite propagation in time determined by the window size. These wavelets are limited in time, whereas sinusoidal functions that are used for the Fourier-based transforms are continuous in the whole time range. Hence, the wavelets are stretched and compressed in frequency. They are also shifted in time to correlate with the original signal under analysis in order to determine the set of frequencies propagating at any instantaneous time moment, following the Heisenberg principle of uncertainty [36]. The WT can be represented by the following equation:

(1) $$x_{\omega }(a,b)=\frac{1}{\sqrt{\left|a\right|}}\int_{-\infty }^{\infty }x(t)\psi^{*}(\frac{t-b}{a})dt$$

where *x(t)* is the target time series, *x_ω_* is the resulting wavelet analysis, ψ* is the complex conjugation of the mother wavelet that has to be a continuous function in both time and frequency domains, *a* is a scale factor that either stretches (large *a*) or compresses (small *a*) the wavelet and, *b* is the signal’s shifting parameter in time. By choosing a different mother wavelet, different characteristics of the time signal can be emphasized as an output. The flexibility of choosing the optimal mother wavelet is one of the advantages of using WT, for the choice of the mother wavelet for a particular problem improves the signal processing capability of the technique. If the shape of the signal to be detected is known a priori, a replica of the set can be utilized as the mother wavelet function, or the mother wavelet can be chosen from a set of theoretical signals. In this regard, the Morlet wavelet is extensively used and ultrasonic signals are investigated due to their behavior which is close to the detected signals. It has been proven to be efficient in improving the signal strength and reducing the noise, making the WT-based technique extremely useful for damage detection [32].

## 3. Materials and Methods

**Figure 1:**
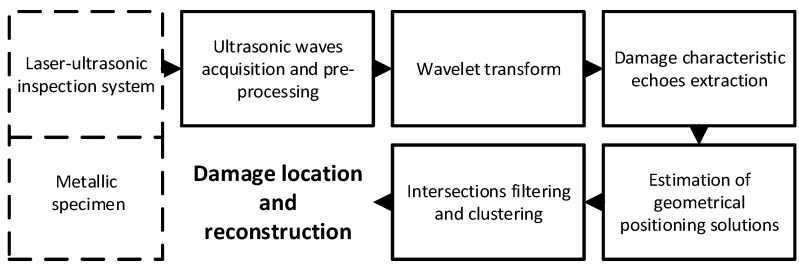
The sequence flow chart of the proposed laser ultrasound imaging methodology composed of five stages.

The proposed laser ultrasound imaging methodology is shown in Figure 1. The methodology consists of five major stages. 

The first stage is the acquisition of ultrasonic signals resulting from the thermal mechanism induced by each considered laser scan point over the specimen scan plane. In this stage, the acquired signals are digitally pre-processed by means of a bandpass filtering in order to increase the resulting signal to noise ratio in the frequency band of interest. 

Second, the wavelet transform is computed for each time-based ultrasonic signal. As aforementioned, a Morlet wavelet is proposed as a good trade-off between performance and simplicity [32]. Thus, it is expected that in the case of internal defect, additional wave fronts at shorter distances than those corresponding to the metallic specimen boundaries appear in the time-frequency response. 

In the third stage of the proposed methodology, it is expected to automatically identify such ultrasonic echoes corresponding to the presence of defect in the considered area of inspection. In this stage, a high-performing clustering algorithm, commonly used in image processing applications, is proposed in order to identify the time instant of eventual ultrasonic echoes over the resulting wavelet-based time-frequency maps. 

The density-based spatial clustering of applications with noise (DBSCAN) [37,38] does not require previous knowledge of the number of clusters, which makes the technique suitable for dealing with multi-modal ultrasonic signals represented in the time-frequency maps. For the purpose of DBSCAN, the time-frequency map points under analysis will be considered as core points, density-reachable ones, or outliers during the clustering procedure. In this regard, the DBSCAN requires only two main parameters, i.e., the distance *ε* corresponding to the maximum radius of the neighborhood to be considered during the analysis of a point *p*, and the minimum number of points to consider a dense region, *p_min_*. 

As a result of this stage, the presence of characteristic damage echoes is detected through the clustering algorithm and its corresponding time is obtained from all scan points. The identified echoes, pertinent to the interaction between the ultrasonic wave front and the defect, correspond to the sum of the TOF between the laser scan point to the defect and back to the ultrasonic transducer. 

In the fourth stage, we automatically identify the corresponding TOFs of the second detected group of echoes by detecting the time of the second cluster. For each resultant TOF, which corresponds to the sum of the individual TOFs from the laser to the defect and the individual TOFs from the defect point to the sensor, an ellipsoid is estimated, as a locus of defect location, which represents a set of possible geometrical solutions matching the computed TOF. An ellipsoid, by definition, is the locus of a point whose sum of displacement from the two well-determined points, the foci of the ellipsoid, is constant. Actually, the proposed method is based on the consideration of discretized three-dimensional curves resulting from the intersections of each pair of ellipsoids. From a geometrical point of view, the embedded defect generating the corresponding time of flight is estimated to be at any point in such a curve. So, every point of the resulting curve is a potential locus of the defect. This continuous three-dimensional intersection curve, when discretized, is converted to points that become the locus of intersection. Thus, considering *N* ellipsoids generated by *N* different measurements resulting from *N* different scan points. A total number of intersections, *I*, considering any pair of ellipsoid intersection will be obtained, following:

(2) $$I=N_{C_{2}}=\frac{N!}{2\times (N-2)!}$$

Finally, the fifth stage proposes the identification of coherent intersections among the ellipsoids resulting from all laser scan point computations through a density analysis. In this stage, it must be noted that a previous filtering is proposed to reject the ellipsoids resulting from TOFs larger than the dimensions of the considered scan area. Then, the re-iterative intersections in the considered scan volume are projected in the scan plane x-y, where the DBscan technique is proposed to extract maximum density areas corresponding to a higher probability of defect location. 

The analysis of the resulting set of ellipsoid intersections is proposed to be faced with the application of the DBSCAN-based clustering algorithm once more in our methodology. Then, the spatial localization of the defect is obtained by the geometrical center calculation among the resulting clustered intersection points, and the defect reconstruction for visualization is carried out by means of the outline of the clustered intersection points.

**Figure 2:**
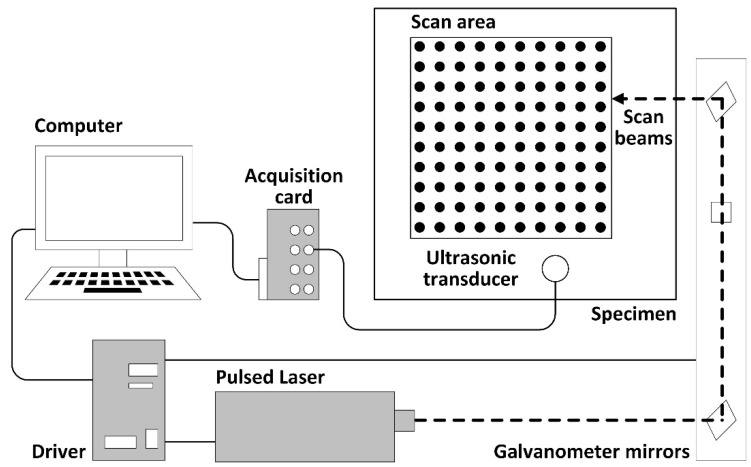
A schematic diagram of the laser ultrasonic inspection system configuration, including the pulsed laser, galvanometer mirrors, acquisition card, and ultrasonic transducer.

The schematic diagram of the entire laser-ultrasonic inspection system configuration, considered for method validation, is shown in Figure 2. The ultrasonic waves are generated by a Nd:YAG pulsed laser from Litron Lasers, model LPY604-10 with 532 nm, 200 mJ, and 10 ns of pulse duration. An external computer controls both the galvanometer mirrors and pulsed laser to shift the laser beam within a predefined scan area. The laser ultrasonic inspection system includes an Olympus V133-RM ultrasonic transducer with a resonance frequency at 2.25 MHz. The 2D scanning mirror galvanometer is a Thorlabs GVS302 (Newton, NJ, USA). The acquisition system is completed with an Olympus preamplifier 5662 (Tokyo, Japan), and a high-performance Gage A/D card model Oscar Express CSE4444 (DynamicSignals LLC, Lockport, IL, USA) that allows a sampling frequency of 50 MHz at 16 bit of resolution.

**Figure 3:**
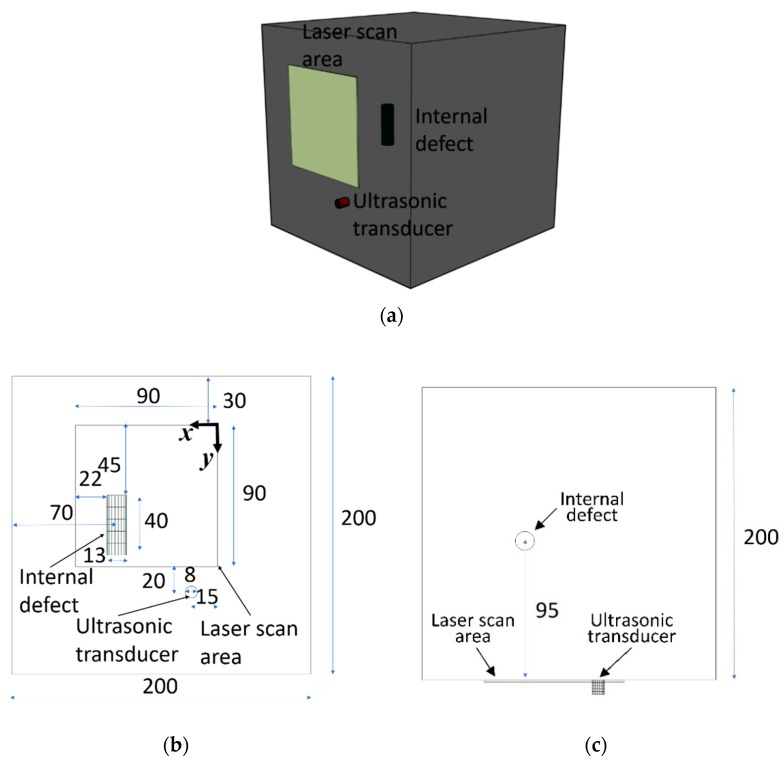
Aluminum specimen, internal damage and laser scan area. All dimensions are in millimeters. (**a**) Isometric view. (**b**) Front view. (**c**) Top view.

As shown in Figure 3, the considered aluminum object to be inspected is 200 mm × 200 mm × 200 mm, with a mechanically induced cylindrical defect sized 40 mm of length and 13 mm of diameter located at 95 mm depth from the specimen’s surface considered during the inspection. The scan area is 90 mm × 90 mm covered by 10 × 10 laser scanning points. The aluminum object is fabricated on a local casting workshop. The aluminum was cast on a prepared cubic model. A piece of hard glass with cylindrical shape was placed inside the model with the dimensions and positions mentioned in Figure 3 to act as the internal defect. The glass was hard solid and temperature resistant to withstand the casting of the hot liquid aluminum. We considered longitudinal wave propagation for this study as it penetrates to the depth of the material. Moreover, our ultrasonic receiver response has higher efficiency at detection of longitudinal waves. The velocity of longitudinal wave propagation in homogenous aluminum is 6300 m/s [5].

## 4. Results

**Figure 4:**
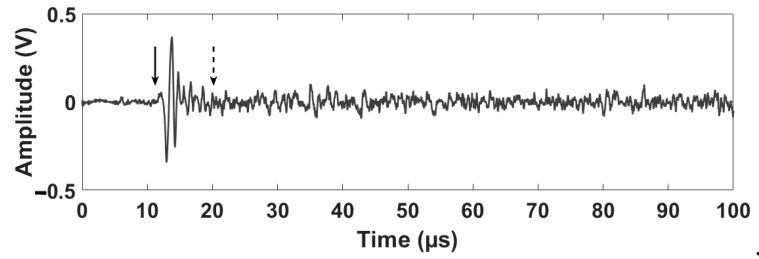
An example of the resulting laser-generated ultrasonic wave experimentally measured over the defective aluminum specimen. The solid arrow indicates the time of the direct laser-induced ultrasonic wave arrival (detected visually), while the dotted arrow indicates the estimated time instant of the ultrasonic defect-reflected echo arrival (detected theoretically). The absolute coordinates corresponding to the laser scan point are *x* = 11 mm and *y* = 51 mm, while the absolute coordinates corresponding to the ultrasonic transducer are *x* = 15 mm and *y* = 110 mm.

An example of the ultrasonic waves resulting from the thermal mechanism induced during the pulsed laser excitation scan is shown in Figure 4. 

The time of the initial ultrasonic wavefront arrival to the ultrasonic sensor in Figure 4 can be detected visually at the time 12.25 µs. Although the damage effect in the time domain of the recorded ultrasonic signal is not detectable, such influence can be expected at time 20.50 µs. This is calculated theoretically as follows [39]:

(3) $$\begin{array}{l} TOF=\frac{\sqrt{{\left(x_{laser}-x_{defect}\right)}^{2}+{\left(y_{laser}-y_{defect}\right)}^{2}+{\left(z_{laser}-z_{defect}\right)}^{2}}}{Longitudonal\;Velocity}+\\ \frac{\sqrt{{\left(x_{sensor}-x_{defect}\right)}^{2}+{\left(y_{sensor}-y_{defect}\right)}^{2}+{\left(z_{sensor}-z_{defect}\right)}^{2}}}{Longitudonal\;Velocity} \end{array}$$

**Figure 5:**
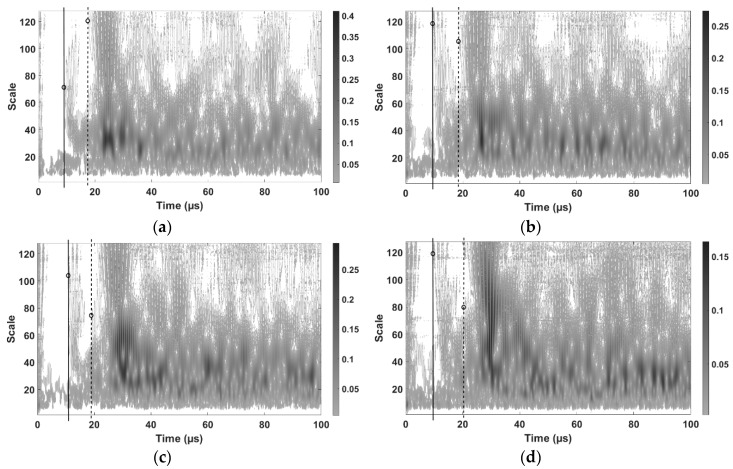
Time-frequency maps of ultrasonic waves induced at different scan points. The dotted line, represents direct ultrasonic wave arrival, the solid line, ultrasonic defect-reflected echo arrival. The corresponding ultrasonic transducer position is *x* = 15 mm and *y* = 110 mm, and the positions of laser scan points are: (**a**) *x* = 11 mm and *y* = 71 mm (**b**) *x* = 11 mm and *y* = 51 mm. (**c**) *x* = 31 mm and *y* = 41 mm. (**d**) *x* = 81 mm and *y* = 41 mm.

In this regard, the corresponding wavelet transform is carried out for each acquired ultrasonic wave during the laser scan process using Morlet mother wavelet. Some resulting time-frequency maps corresponding to ultrasonic waves induced in the aluminum specimen at different laser scan points are shown in Figure 5.

Figure 5 shows how the first significant wavefront comes at the early stages due to the direct surface waves from the laser source to the ultrasonic receiving sensor. Also, as mentioned above, the relative distance between the scan point to the object boundaries and back to the sensors is larger than that from the considered scan point to the defect and back to the sensor. So, the second group of detected ultrasonic waves corresponds to echoes due to the existence of damage. The echoes corresponding to the boundaries are much more delayed as their distance is much larger. In this regard, Figure 5a shows the first wave front arrival, as indicated by circles on solid lines, at 8.8 µs. In Figure 5b, it is at 9.4 µs, in Figure 5c, it is at 10.9 µs and, in Figure 5d, it is at 9.5 µs. Similarly, a second wave front corresponding to the damage presence, as indicated by circles on dashed lines, appears in Figure 5a at 17.4 µs, in Figure 5b at 18.6 µs, in Figure 5c at 19.6 µs and, in Figure 5d at 20.2 µs. Such reflected waves correspond to characteristic damage ultrasonic echoes and specifically their corresponding TOF are considered next to estimate the potential localizations of the defect position.

**Figure 6:**
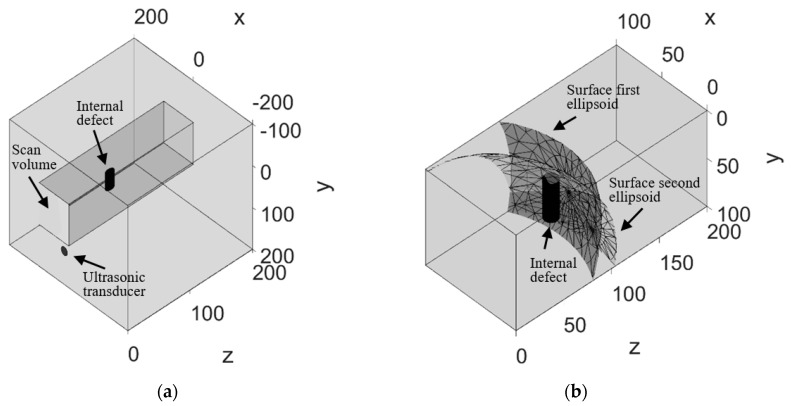
Resulting ellipsoid intersections from two-second echo TOFs estimated during the proposed analysis of two laser-generated ultrasonic waves experimentally measured. (**a**) Isometric view of the scan volume allocation. (**b**) Details of the scan volume and the intersection of two resulting ellipsoids and its coherence with regard to the defect position. The first ellipsoid results from the ultrasonic transducer’s position, scan point 1 position, and TOF 17.4 mm. The second ellipsoid results from the ultrasonic transducer’s position scan point 2 position, and TOF 18.6 mm.

As an example, Figure 6 shows the ellipsoid resulting from the defect location estimation based on two measurements corresponding to different scan positions.

Considering all 10 × 10 ellipsoid estimations the intersections among them correspond to potential locations where the interaction between the laser-generated ultrasonic wave and the defect took place during the inspection. In this regard, Figure 6 shows the details related to the resulting intersection between the ellipsoids corresponding to the two scan points and the relevant TOF of the second echoes.

**Figure 7:**
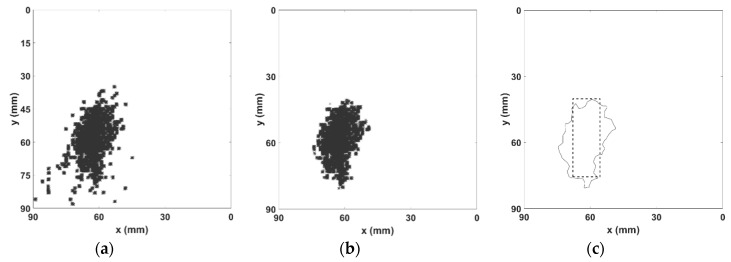
The recurrent intersections set resulting from the ellipsoids analysis. (**a**) The raw set of recurrent ellipsoid intersections. (**b**) The clustered set of ellipsoid intersections. (**c**) The resulting defect reconstruction boundary with the superimposed actual cylindrical defect projection.

Figure 7a shows the complete resulting intersections among all ellipsoids, where only a recurrent filtering is applied. The proposed application of the clustering algorithm, the DBSCAN, over such a resulting set of intersections results in a refined set as shown in Figure 7b, where the resulting intersections after the clustering analysis is presented.

In Figure 7c, we only show the boundary of the resulting clustered set with the superposition of the actual defect location in order to compare the results with the exact position of the defect. The application of the proposed method results in a geometrical defect location estimation centered at *x* = 62 mm and *y* = 69.5 mm, while the actual defect location center is at *x* = 61.5 mm and *y* = 65 mm, thus, revealing a positioning error of 4.53 mm. Considering the size of the defect, the proposed methodology results in an estimation of 578 mm^2^, while the actual dimension of the defect’s cross section area is 520 mm^2^, thus, resulting in an error of 11.1%.

## 5. Conclusions

This paper presents a novel methodology to detect, quantify, and reconstruct internal defects on metallic samples based on laser-generated ultrasound. There are three important aspects of this new method. The first one is the ultrasonic wave signal analysis through wavelet processing. The multi-resolution capabilities of the wavelet analysis allow the identification of the main ultrasonic modes during the front wave propagation from the excitation point to the defect, and finally to the sensor; thus, enhancing characteristic echo detection for each point considered in the scan. The second is the extraction of characteristics of damage echoes and the methodological estimation of ellipses through the resulting time of flight for each laser excitation point. The third is the application of a clustering strategy as a processing stage in order to enhance the significance of the intersection analysis among ellipses. The application of the proposed clustering method over the resulting points of intersection among ellipses allows the identification of the most coherent region and elimination of the less significant intersections. An internal defect at 95 mm from the surface of inspection is considered, which represents a challenging condition. Under such a scenario, the proposed methodology shows reliable fault diagnosis results, with a location and sizing error of 4.53 mm and 11.1%, respectively. The results obtained in this work suggest that this methodology may be also useful for further 3D imaging reconstruction of defects. However, the three-dimensional reconstruction will require a further analysis of multiple echo intersections in order to estimate the depth of the defect. The future work will focus on the analysis of the proposed laser ultrasound imaging method considering boundary interaction within the scan area.
